# Clinical impact of postoperative loss in psoas major muscle and nutrition index after radical cystectomy for patients with urothelial carcinoma of the bladder

**DOI:** 10.1186/s12885-017-3231-7

**Published:** 2017-03-31

**Authors:** Makito Miyake, Yosuke Morizawa, Shunta Hori, Nagaaki Marugami, Keiji Shimada, Daisuke Gotoh, Yoshihiro Tatsumi, Yasushi Nakai, Takeshi Inoue, Satoshi Anai, Kazumasa Torimoto, Katsuya Aoki, Nobumichi Tanaka, Kiyohide Fujimoto

**Affiliations:** 1 Department of Urology, Nara Medical University, 840 Shijo-cho, Kashihara-shi, Nara, 634-8522 Japan; 2 Department of Radiology, Nara Medical University, 840 Shijo-cho, Kashihara-shi, Nara, 634-8522 Japan; 3 Department of Pathology, Nara City Hospital, 1-50-1 Higashi kidera-cho, Nara-shi, Nara, 630-8305 Japan

**Keywords:** Skeletal muscle index, Sarcopenia, Prognostic nutritional index, Controlling nutritional status, Bladder cancer, Radical cystectomy, Prognosis

## Abstract

**Background:**

Although the significance of preoperative nutritional status has been investigated, there is no report regarding the relationship of their postoperative changes on outcomes in patients who underwent radical cystectomy for bladder cancer. Here, we report the clinical impact of the change, from baseline, in nutritional status and volume of abdominal skeletal muscle mass and adipose tissue after radical cystetomy.

**Methods:**

A retrospective analysis of 89 patients with bladder cancer, who underwent curative radical cystectomy, was conducted to assess the time course of change, from baseline, in body composition and nutritional status at 1, 3, 6, 12, and 24 months, after surgery. Skeletal muscle mass and abdominal adipose tissue mass were quantified by unenhanced computed tomography images. Two different nutritional indices, the Prognostic Nutritional Index and the Controlling Nutritional Status score were calculated from laboratory blood tests. We evaluated the prognostic value of the rate of change in the body composition and nutritional status after radical cystectomy.

**Results:**

The cross-sectional area at the level of the third lumbar vertebra of the psoas major muscle and nutritional indices showed a transient deterioration at 1 and 3 months after radical cystectomy, with a return to baseline values from 6 to 24 months. A ≤ −10% loss in the area of the psoas muscle was associated with a shorter overall survival, compared to those with a > −10 change [hazard ratio (HR) 2.2, *P* = 0.02]. Multivariate analyzes identified sarcopenia status at baseline (HR 2.2, *P* = 0.03) and a ≤ −10% loss in the psoas muscle (HR 2.4, *P* = 0.02) were identified as independent prognostic factors for overall survival. A subanalysis of patients *without* sarcopenia identified a worse survival outcome for patients with a ≤ −10% loss in the psoas muscle (HR 2.6, *P* = 0.03) and ≤ − 5 change in the Prognostic Nutritional Index (HR 3.6, *P* = 0.01).

**Conclusion:**

Further research is required to establish appropriate rehabilitation protocols and nutritional interventions after radical cystectomy for maintaining skeletal muscle mass and nutrition status which could counteract physical deterioration and improve outcomes.

**Electronic supplementary material:**

The online version of this article (doi:10.1186/s12885-017-3231-7) contains supplementary material, which is available to authorized users.

## Background

Urothelial carcinoma of the bladder (UCB) is the sixth most frequent neoplasm in men [1]. Radical cystectomy (RC), with or without perioperative systemic chemotherapy, is the standard treatment for selected T1 high-grade and muscle invasive bladder cancer (≥T2; MIBC) [2, 3]. Both postoperative complication and mortality are significant concerns in these patients. Simple and specific factors predictive of postoperative complications and mortality are required to improve clinical outcomes after RC.

Over the past decade, studies have evaluated the relationship between postoperative complication and/or mortality and several preoperative factors, including body mass index (BMI), nutritional status, inflammation, host immunity, skeletal muscle mass (i.e.*,* sarcopenia) and abdominal adipose tissue, in various malignant diseases, including bladder, upper urinary tract, kidney, prostate, colorectal, and hepatocellular carcinoma [4–14]. Specifically with regards to the effects of sarcopenia on postoperative outcomes, the clinical definition of age-related sarcopenia developed by The European Working Group on Sarcopenia in Older People (EWGSOP) in 2010 has been used, which bases the diagnosis of sarcopenia on documentation of low muscle mass, low muscle strength and low physical performance [15].

Loss of muscle mass can easily be evaluated using abdominal computed tomography (CT), which is routinely performed as part of an oncological evaluation. Therefore, majority of studies have evaluated the impact of baseline measures in skeletal muscle mass and physical function, that were obtained before surgery and/or oncological treatment, on postoperative complications, prognosis and treatment outcomes in patients after systemic chemotherapy or radical surgery [7, 8, 12, 16–19]. To date, however, the associations of change, from baseline, in skeletal mass and adipose tissue on prognosis and treatment outcomes have not been evaluated in patients with UBC undergoing RC.

As concurrent urinary diversion requiring intestinal anastomosis, such as ileal conduit and orthotopic neobladder, is performed as part of an RC, an accurate evaluation of preoperative nutritional status is an essential component of the preoperative evaluation. The preoperative immune-nutritional status, including the prognostic nutritional index (PNI) [20] and the Controlling Nutritional Status (CONUT) score [13], has been reported to correlate with survival and postoperative complications in patients with colorectal cancer. These scores are calculated from the serum albumin, the peripheral lymphocyte count and total cholesterol (T-chol). Although the clinical relevance of the PNI and CONUT has previously been investigated in other malignancies [13, 20], there is no report regarding the relationship of these preoperative and postoperative nutritional scores on outcomes after RC. Therefore, the aim of our study was to perform a comprehensive analysis of the change, from baseline, nutritional status, volume of abdominal skeletal muscle mass/adipose tissue and its effect on the postoperative prognosis of patients who underwent RC for UCB.

## Methods

### Patient selection and data collection

Between January 2006 and October 2014, 105 UCB cases, without evidence of distant metastases, underwent curative RC. Sixteen cases were excluded because of insufficient radiographic and laboratory examination, leaving the data from 89 cases to be included in our retrospective analysis. The histopathological review was conducted by an experienced uropathologist (KS) to determine the T category (2010 AJCC TNM Staging system), tumor grade (2004 WHO classification) and presence of variant histology. Clinicopathological variables and laboratory data obtained at baseline and at all time points of measurement postoperatively were extracted from medical records for analysis of the time-course of change after surgery. Among the 89 cases, 37 (42%) and 14 (16%) received 2 or 3 cycles of platinum-based neoadjuvant chemotherapy and adjuvant chemotherapy, respectively.

### Body composition index by CT scan

**Fig. 1:**
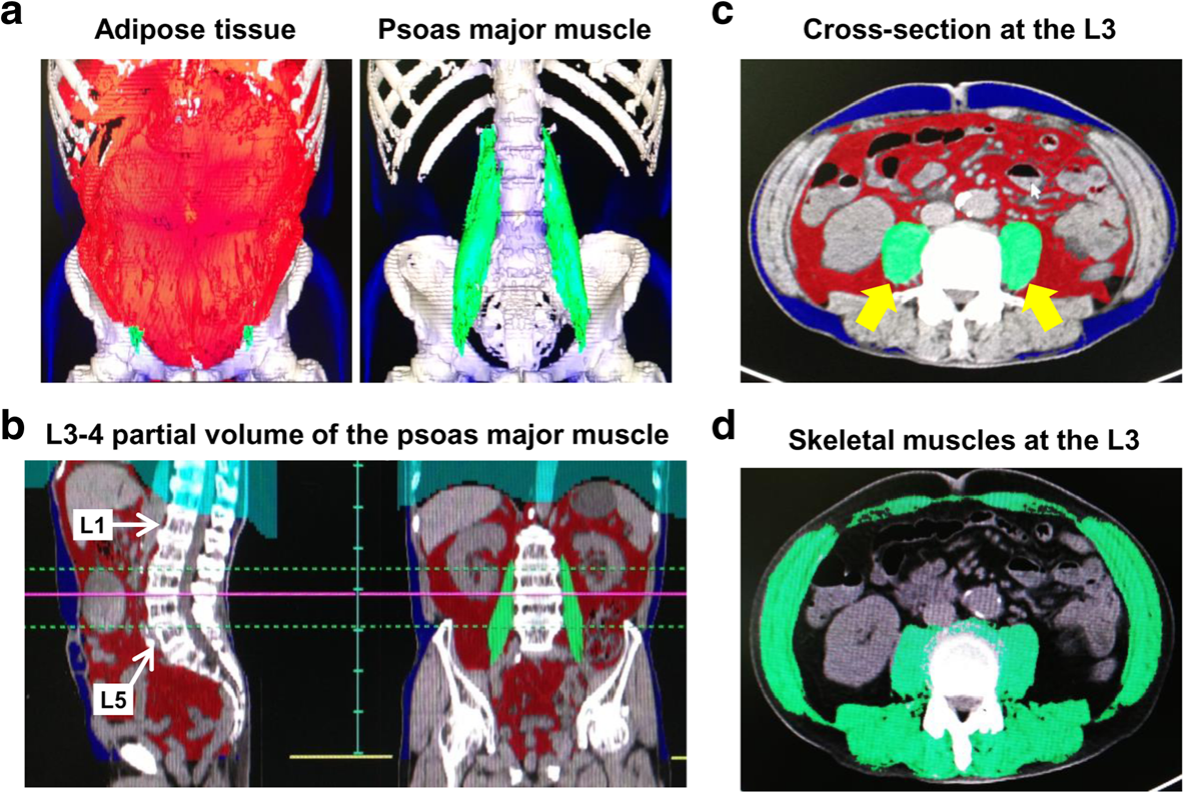
Representative images for the body composition indexVolume Analyzer SYNAPSE VINCENT image analysis system was used to reconstruct three-dimensional (3-D) images as follow: **a**, red area indicates abdominal adipose tissue (visceral fat and subcutaneous fat), with the green area indicating the total volume of the psoas major muscle; **b**, quantification of the partial volume of the psoas major muscle at the level of L3-L4; **c**, cross-sectional area of the psoas major muscle at the level of L3 (green ovals indicated by yellow arrows); and **d**, cross-sectional 2-D images at the level of L3 were used to quantify the skeletal muscle index (SMI). The green shadows indicate the abdominal skeletal muscle areas

Unenhanced CT images, performed for diagnostic or follow-up purposes, were used as inputs into the Volume Analyzer SYNAPSE VINCENT image analysis system (Fujifilm Medical, Tokyo, Japan) to quantify the body composition index. Measured variables were normalized to height (m^2^) and were expressed in cm^3^/m^2^ or cm^2^/m^2^ for between-subject comparisons. The following measures were obtained for analysis: total psoas major muscle volume (cm^3^/m^2^); partial volume of the psoas major muscle at the level of the third and fourth lumbar vertebra (L3-L4; cm^3^/m^2^); area of the psoas major muscle at L3 (cm^2^/m^2^); total area of the abdominal skeletal muscle (cm^2^/m^2^); and the volume of abdominal adipose tissue, commonly referred to as the volume of subcutaneous and visceral fat (cm^3^/m^2^). The visceral-to-subcutaneous adipose tissue volume ratio (VSR) was calculated to determine the distribution of abdominal adipose tissue. Representative images used for analyses of abdominal muscle and fat are shown in Fig. 1a-c.

### Skeletal muscle index and definition of sarcopenia

Abdominal skeletal muscles include the psoas major, paraspinals (erector spinae and quadratus lumborum), and muscles of the abdominal wall (transversus abdominus, external and internal obliques, and rectus abdominus). The cross-sectional area of skeletal muscle at the level of L3 (cm^2^), which was identified using Hounsfield unit thresholds of −30 to +150, was normalized to height (m^2^) to yield the skeletal muscle index (SMI), expressed in cm^2^/m^2^ (Fig. 1d) [16]. Image analysis was performed by two independent investigators (MM and YM) who were blinded to other parameters and outcomes at the time of measurement. Martin’s definition of the SMI [21] was adopted in our analysis as it is based on a large cohort and, therefore, would be applicable to various cancer patients with different BMI profiles, as follows: SMIs of <43 cm^2^/m^2^ for males with a BMI <25 kg/m^2^; <53 cm^2^/m^2^ for males with a BMI ≥25 kg/m^2^; and <41 cm^2^/m^2^ for females.

### Nutrition index

The baseline blood data were obtained the day before RC. The PNI was calculated using the following formula: 10 × serum albumin (g/dl) + 0.005 × total lymphocyte count (per mm^3^) [18]. The CONUT score was determined on the basis of the serum albumin, peripheral lymphocyte count and the T-cholesterol, as previously described (Table 1) [22]. Both indices were reported as continuous variable for analysis. Cutoffs for the PNI and CONUT scores were set to the lower quartile and higher quartile values among the study group, respectively, based on the interquartile range (IQR).

**Table 1:** Scoring table for the CONUT score

Variables	Malnutrition degree			
	None	Mild	Moderate	Severe
Serum albumin (g/dL)	≥ 3.5	3.00–3.49	2.50–2.99	< 2.5
Score	0	2	4	6
Total lymphocyte count (/mm3)	≥ 1600	1200–1599	800–1199	< 800
Score	0	1	2	3
Total cholesterol (mg/dL)	≥ 180	140–179	100–139	< 100
Score	0	1	2	3

CONUT = controlling nutritional status; cited from de Ulibarri Perez JI, et al. Nutr Hosp [22]

### Follow-up and time-course of change in variables after RC

Postoperative follow-up was performed at the following time points, according to our institutional protocol: 1 month, every 3 months for the first 2 years, every 6 months for the following 3 years, and annually thereafter for patients without evidence of recurrent disease [23]. Oncological evaluation included physical examination, routine blood tests, urine cytology, and CT scan of the chest, abdomen and pelvis. Body composition variables and nutritional index were retrospectively determined and their postoperative time-course change, from baseline, was calculated by absolute value and relative value analysis, with baseline data set to 100%.

### Statistical analysis

Data were expressed by bar charts or box plots, and evaluated using Student’s *t*-test or the Mann–Whitney *U*-test as appropriate for the dataset. Disease-specific survival (DSS) and overall survival (OS) were estimated using the Kaplan–Meier method and compared using the log-rank test or log-rank test for trend analysis. Multivariate analysis was used to identify independent prognostic variables using a stepwise Cox proportional hazards regression model. Variables significantly affecting mortality (*P* < 0.05) in univariate analysis were included in the multivariate analysis. IBM SPSS Version 21 (SPSS Inc., Chicago, IL, USA) and PRISM software version 5.00 (San Diego, CA, USA) were used for statistical analyzes and plotting the data, respectively. A *P* value <0.05 was considered statistically significant.

## Results

### Patient characteristics and the relationship with sarcopenia

The baseline clinicopathological variables, nutritional index, blood test results, and the administration of neoajuvant/adjuvant chemotherapy for the 89 cases included in our analysis are summarized in Table 2. The median follow-up period after RC for the survival analysis was 29 months (IQR, 10–60). Over the follow-up period, 35 patients (39%) died, 25 (28%) of UCB. The mean ± SD (cm^2^/m^2^) SMI before RC in men and women, respectively, was 52.1 ± 11.1 and 46.9 ± 7.6. Twenty two patients (25%) were diagnosed with sarcopenia in our study cohort. Baseline variables were compared between patients *with* and *without* sarcopenia (Table 2). Sarcopenia status was associated to a lower BMI and clinical T4 category, while there was a marginal association of sarcopenia status with a lower PNI, a lower hemoglobin, and a lower sodium.

**Table 2:** Characteristics and correlation with sarcopenia in 89 patients undergoing radical cystectomy

Variables	Total cases, n (%)	Non-sarcopenia, n (%)†	Sarcopenia, n (%) ^†^	*P value ¶*
Total cases	89 (100%)	67 (75%)	22 (25%)	-
Age at RC (year-old), median (IQR)	71 (48–83)	72 (66–75)	71 (62–76)	0.22
BMI (Kg/m^2^), median (IQR)	22.7 (20.9–24.7)	23.3 (21.6–24.75)	21.8 (19.3–23.0)	0.013
Sex				0.16
Male	70 (79%)	55 (82%)	15 (68%)	
Female	19 (21%)	12 (18%)	7 (32%)	
ECOG-PS				1.0
0	82 (92%)	62 (93%)	20 (91%)	
≥ 1	7 (8%)	5 (7%)	2 (9%)	
Clinical T category				0.007
NMIBC	6 (7%)	2 (3%)	4 (18%)	
T2	42 (47%)	36 (54%)	6 (27%)	
T3	19 (21%)	16 (24%)	3 (14%)	
T4	22 (25%)	13 (19%)	9 (41%)	
Clinical N category				0.83
0	78 (88%)	59 (88%)	19 (86%)	
≥ 1	11 (12%)	8 (12%)	3 (14%)	
Variant histology				0.21
No	68 (76%)	49 (73%)	19 (86%)	
Yes	21 (24%)	18 (27%)	3 (14%)	
Preoeprative nutrition index, median (IQR)				
PNI	47.0 (43.5–50.2)	47.5 (44.0–51.0)	45.2 (38.8–48.6)	0.08
CONUT score	2 (1–3)	2 (1–3)	2 (1–3)	0.30
Preoeprative blood level, median (IQR)				
Hb (g/dL)	11.2 (10.2–12.7)	11.6 (10.4–12.7)	10.8 (9.8–12.1)	0.07
CRP (mg/dL)	0.2 (0.1–0.7)	0.2 (0.1–0.6)	0.4 (0.1–1.7)	0.20
Albumin (g/dL)	4.0 (3.7–4.2)	4.0 (3.8–4.2)	3.9 (3.6–4.2)	0.47
Sodium (mEq/L)	141 (139–143)	141 (139–143)	140 (138–143)	0.09
LDH (U/L)	180 (159–201)	180 (159–201)	181 (139–205)	0.54
Neoadjuvant chemotherapy				0.23
No	52 (58%)	38 (57%)	14 (64%)	
Yes	37 (42%)	29 (43%)	8 (36%)	
Adjuvant chemotherapy				0.42
No	75 (84%)	56 (84%)	19 (86%)	
Yes	14 (16%)	11 (16%)	3 (14%)	

*RC radical cystectomy*; *IQR* interquartile range; *ECOG-PS* Eastern Cooperative Oncology Group-Performance Status, *NMIBC* non-muscle invasive bladder cancer (T1/isolated Tis), *BMI* body mass index, *PNI* prognostic nutritional index, *CONUT* controlling nutritional status, *Hb* hemoglobin, *CRP* C-reactive protein *LDH* lactate dehydrogenase

^†^ According to the definition for the Martin's definition [21]

¶ Comparison between non-sarcopenia cases and sarcopenia cases female with Mann-Whitney U test or chi-squared test

### The baseline body composition and nutritional indices

The median (IQR) and mean ± SD of the abdominal muscle index, the abdominal adipose tissue index, and the nutritional indices at baseline are listed in Additional file 1: Table S1. Although there was no sex-specific difference in BMI, the visceral fat index, PNI and CONUT score, significant sex-specific differences were identified for all abdominal muscle indices with higher abdominal muscle indices in males, with the subcutaneous fat index and VSR being higher in females. Based on this result, relative values (expressed as a percentage of the baseline, which was set at 100%) were more appropriate to plot the data of male and female patients all together in the analysis of the postoperative change, from baseline, in body composition index than absolute values.

### Postoperative time course of change in body composition and nutritional indices

**Fig. 2:**
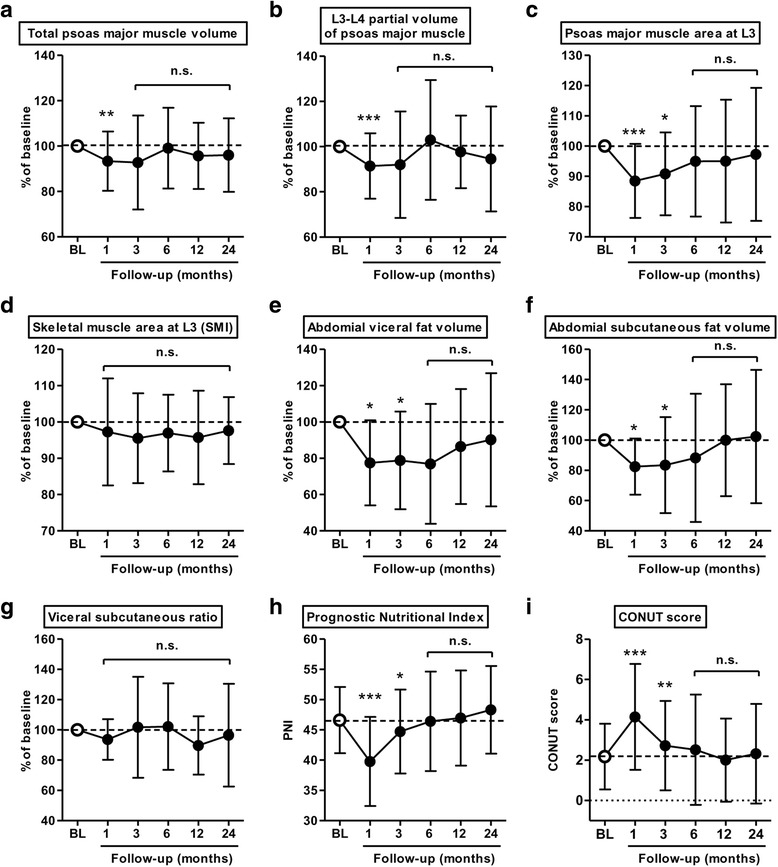
Time course of change in the body composition and nutrition indices after radical cystectomy. **a**, total volume of the psoas major muscle; **b**, partial volume of the psoas major muscle at the level of L3-L4; **c**, cross-section area of the psoas major muscle at the level of L3; **d**, abdominal skeletal muscle area at the level of L3, referred as skeletal mass index (SMI); **e**, volume of abdominal visceral fat; **f**, volume of abdominal subcutaneous fat; **g**, visceral-to-subcutaneous ratio (fat distribution); **h**, the PNI; and **i**, the CONUT score. Relative values are used for the body composition indices (**a-g**), and raw values for nutrition indices (**h** and **i**). For the body composition indices, the time course of change in absolute values is shown in Fig. S1. Data are expressed as means and standard deviations. Scores at each time point (1, 3, 6, 12, and 24 months after radical cystectomy) were compared to baseline (BL) scores using the Wilcoxon signed-rank test: *, *P* < 0.05; **, *P* < 0.01; and ***, *P* < 0.001

The change from baseline to 24 months after RC was plotted on line graphs (Fig. 2a-h), with absolute values shown in Additional file 2: Figure S1. A transient deterioration was identified for most variables at 1 and 3 months after RC, with the majority of scores recovering to baseline values over time. Although the SMI (all abdominal skeletal muscle volume at L3) exhibited little deterioration (Fig. 2d), the decrease in the psoas major muscle area at L3 remained significant at 3 months (Fig. 2c). A similar trend was seen in changes of two abdominal fat index and VSR (Fig. 2e-g).

**Fig. 3:**
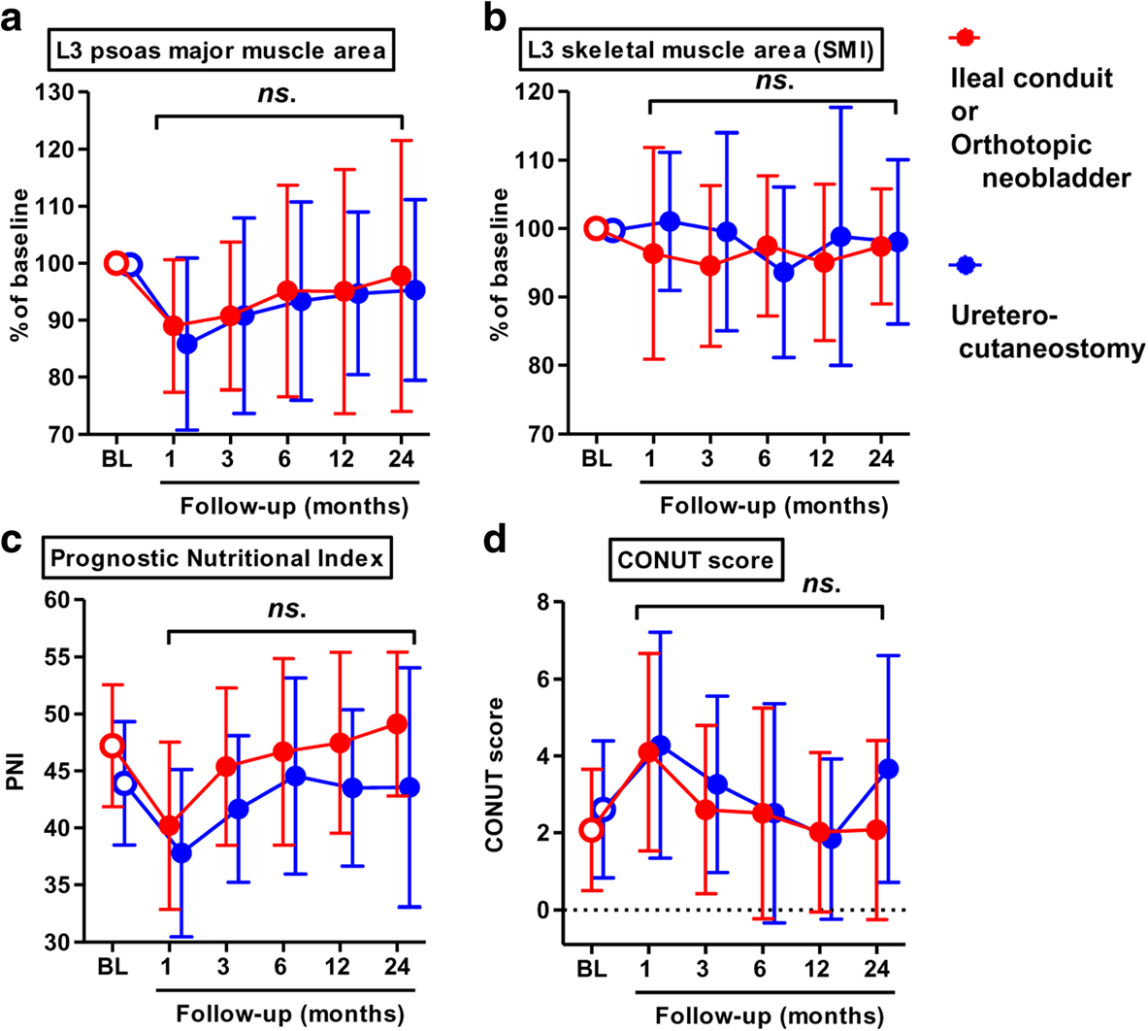
Comparison of changes after radical cystectomy between ileal conduit/neobladder group and uretero-cutaneostomy group.Time-course changes in cross-section area of the psoas major muscle at the level of L3 (**a**), abdominal skeletal muscle area at the level of L3 (**b**), the PNI (**c**) and, the CONUT score (**d**). Data of ileal conduit/neobladder group (red) and cutaneostomy group (blue) conduit are plotted by means ± SD. Scores of two groups compared in each time point by the Mann-Whitney U-test. ns, not significant

Of 89 patients, 71 (80%) underwent ileal conduit, 1 (1%) underwent orthotopic ileal neobladder, and 17 (19%) underwent uretero-cutaneostomy. The former two requires intestinal anastomosis, while the latter does not. To test whether urinary diversion method affects time-course changes in skeletal muscle volume and nutrition status, we compared their changes between ileal conduit/neobladder and cutaneostomy (Fig. 3). Similar analyses were performed for the administration of adjuvant and neoadjuvant chemotherapy (Additional file 3: Figure S2 and Additional file 4: S3). However, none of urinary diversion method, adjuvant chemotherapy, and neoadjuvant chemotherapy affected significantly the changes in skeletal muscle volume and nutrition status.

**Fig. 4:**
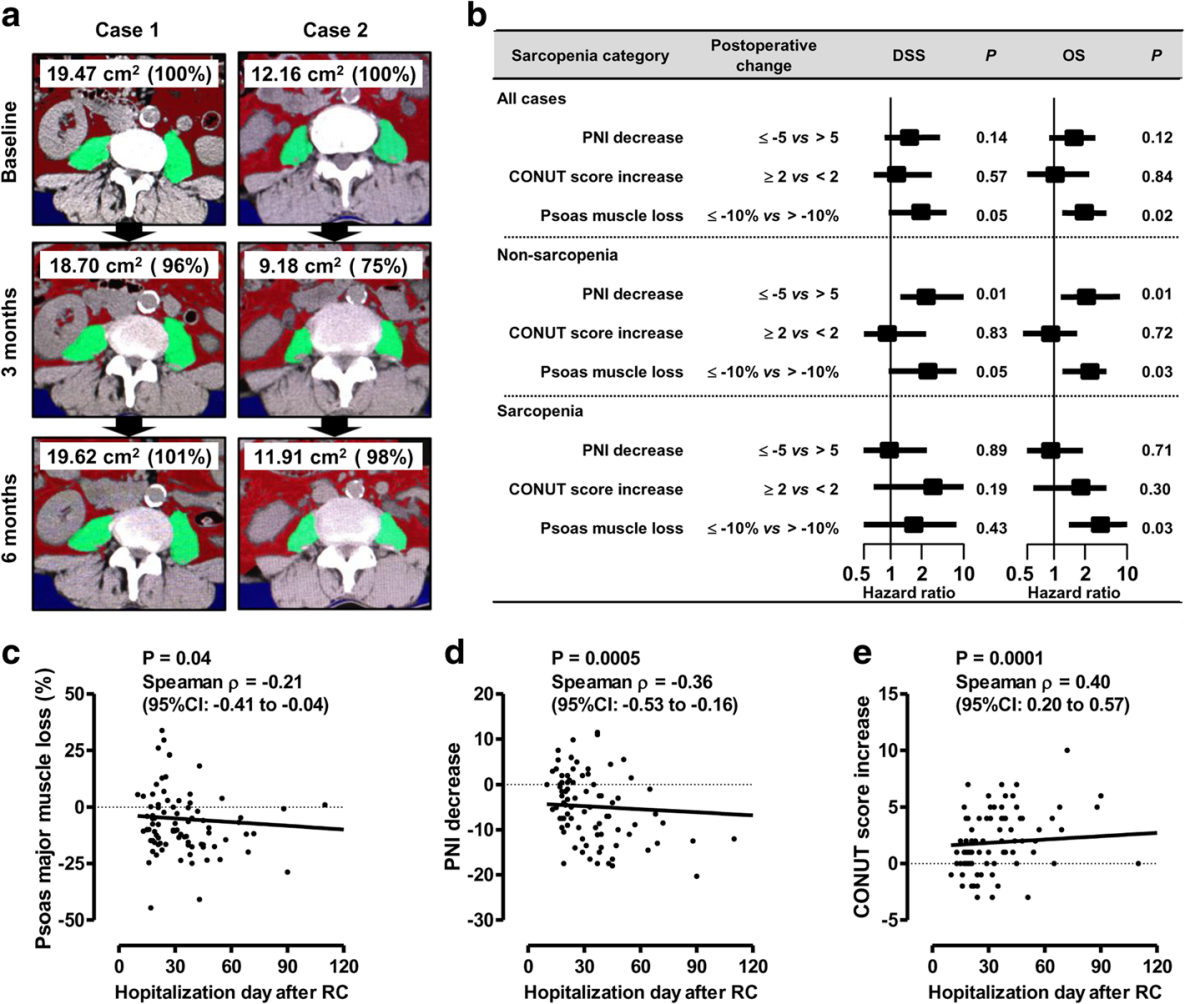
Prognostic impacts of postoperative psoas muscle loss and nutritional deterioration. **a**. Time course of change (from baseline to 3 and 6 months) in the area of the psoas major muscle at the level of L3, on CT images, is shown for two representative patients. Case 1 (60 year-old man diagnosed with pT2a high-grade UC with nested variant) showed a 4% loss in muscle volume. There was no evidence of recurrence of metastases at 80 months after radical cystectomy. Case 2 (72 year-old man with pT3a high-grade pure UC) showed a 25% loss in muscle volume. A metastatic lesion was detected in the ileum at 31 months after cystectomy, and the patient died of bladder cancer at 54 months after radical cystectomy**b**. Impact of postoperative nutritional deterioration and psoas major muscle loss evaluated across all patients. A subanalysis according to sarcopenia status at baseline was performed. Hazard ratio and 95% confidence interval are plotted in the Table. *P* values are based on the comparison using the log-rank test. PNI, prognostic nutrition index; CONUT, Controlling Nutritional Status; DSS, disease-specific survival; OS, overall survival.**c-e**. The correlation between days of hospitalization after RC postoperative loss in volume of the psoas major muscle (**c**), decrease in PNI (**d**), and increase in CONUT score (**e**). The correlation was examined using Spearman’s correlation; Spearman ρ values and the 95% confidence intervals are shown in the Figures.

Both nutritional indices showed a significant transient deterioration at 1 and 3 months after RC (Fig. 2h and i). We hypothesized that the extent of the partial volume loss of the psoas major muscle at L3 and the deterioration in nutritional indices after RC would be associated with a worse prognosis, as previously reported in a cohort of patients with colorectal cancer and lung cancer treated with chemotherapy [16, 19]. The postoperative time course of change in the area of the psoas major muscle area at L3 is shown in Fig. 4a for two representative patients. Median (IQR) points of maximal change during 1–3 months from baseline for the partial volume of psoas major muscle at L3 and for the PNI and CONUT score were −10.0% (−44.6 to −0.1), −5.0 (−20.3 to −0.3) and +2 (−3 to +4), respectively. The cutoff values for change after RC were determined based on median values as follows: −10% (partial psoas volume), −5 points (PNI) and 2 points (CONUT score). Patients experiencing a ≤ −10% loss in the volume of the psoas muscle after RC had a significantly shorter OS, whereas a change of ≤ − 5 on the PNI and ≥2 on the CONUT score was not associated with a worse survival outcome (Fig. 4b). In a subanalysis of patients *without* sarcopenia (*n* = 67), DSS and OS were significantly worse in patients experiencing a ≤ −5 change in the PNI, compared to those with > − 5 change in PNI (Fig. 4b). A ≤ −10% loss in the volume of the psoas muscle was associated with a shorter OS in all analyzes, including the entire cohort and subanalyzes for patients *with* and *without* sarcopenia (Fig. 4B). No prognostic effect of the CONUT score was identified. The Kaplan–Meier curves for DSS and OS are shown in Additional file 5: Figure S4.

We also evaluated the correlation between the number of days of hospitalization after RC and postoperative loss in psoas major muscle volume, decrease in the PNI and increase in the CONUT score (Fig. 4c-e). Both postoperative muscle loss and deterioration in nutritional indices were significantly related to a longer duration of hospitalization after RC.

### Multivariate analysis for prognostic impact of psoas muscle loss

To explore the prognostic impact of preoperative sarcopenia status, as well as of the postoperative volume loss of psoas muscle, we performed univariate analyzes followed by multivariate analysis (Table 3). Univariate analysis identified T4 category, N positive and sarcopenia as prognostic variables for DSS, with T4 category, N positive, sarcopenia, and postoperative volume loss of the psoas muscle identified as prognostic factors of OS. Multivariate analysis, controlling for possible confounding factors, identified positive N [hazard ratio (HR) 3.1, *P* = 0.02] as an independent prognostic factor of DSS, and sarcopenia status at baseline (HR 2.2, *P* = 0.03) and a 10% loss in the volume of the psoas muscle (HR 2.4, *P* = 0.02) as an independent prognostic factor of OS. Neither sarcopenia status at baseline nor a 10% loss in the volume of the psoas muscle were identified as independent prognostic factors for DSS in this study (*P* = 0.14 and 0.11, respectively).

**Table 3:** Univariate and multivariate analysis of prognostic variables in 89 cases undergoing radical cystectomy

Variables	Disease-specific survival						Overall survival					
	Univariate			Multivariate			Univariate			Multivariate		
	HR	95% CI	*P* value	HR	95% CI	*P* value	HR	95% CI	*P* value	HR	95% CI	*P* value
Age at RC												
≤ 70	1						1					
> 70	1.9	0.8–4.2	0.12				1.7	0.8–3.3	0.15			
Sex												
Male	1						1					
Female	1.4	0.5–3.8	0.52				1.3	0.5–3.0	0.57			
Clinical T												
NMIBC	1			1			1			1		
T2	3.1	0.2–39.7	0.38	0.8	0.09–7.0	0.84	1.3	0.2–8.2	0.8	1.3	0.2–11.5	0.78
T3	3.5	0.2–54.0	0.36	0.7	0.08–6.7	0.72	1.1	0.2–9.2	0.9	0.97	0.2–4.9	0.98
T4	4.0	1.2–13.7	0.03	4.1	0.5–35.4	0.20	3.1	1.1–8.8	0.04	6.7	0.8–56.9	0.08
Clinical N												
N0	1			1			1			1		
N+	11.3	2.8–45.7	0.0006	3.1	1.2–7.8	0.02	3.9	1.2–12.5	0.02	1.9	0.8–4.5	0.15
Variant histology												
Negative	1						1					
Positive	0.9	0.3–2.2	0.76				0.9	0.4–1.9	0.73			
Neoadjuvant chemotherapy												
No	1						1					
Yes	0.7	0.3–1.5	0.35				0.7	0.3–1.5	0.35			
Adjuvant chemotherapy												
No	1						1					
Yes	1.8	0.6–5.3	0.28				0.7	0.4–1.4	0.72			
Sarcopenia status ^†^												
No	1			1			1			1		
Yes	2.7	1.1–7.2	0.048	1.9	0.8–4.5	0.14	2.3	1.1–5.3	0.046	2.2	1.1–4.6	0.03
Psoas major muscle loss												
> −10%	1			1			1			1		
≤ −10%	2.2	0.99–4.8	0.052	2.0	0.9–4.7	0.11	2.2	1.2–4.4	0.02	2.4	1.2–4.9	0.02

*HR* hazard ratio, *CI* confidence interval, *RC* radical cystectomy, *NMIBC* non-muscle invasive bladder cancer (T1/isolated Tis)

^†^ According to the definition for the Martin's definition [21]

## Discussion

There is accumulating evidence supporting the relevance of host immunity, response and inflammation to tumor progression and disease prognosis [24]. Sarcopenia is a multifactorial syndrome that is defined as a loss of skeletal muscle mass and low physical performance [15]. Skeletal muscle loss is a critical physiological change underlying wasting and frailty, resulting in poor clinical outcomes for acute illness and malignant diseases [8, 9, 24]. In addition to wasting of skeletal muscle mass, nutritional status and body composition, including the volume of adipose tissue, have been related with worse clinical and disease prognosis [11, 13, 14, 20, 21]. In our study, we investigated the clinical relevance of baseline and postoperative time course of change in body composition and nutritional indices as prognostic biomarkers to predict DSS and OS in patients with UCB undergoing RC.

Baseline sarcopenia, defined by a low SMI, was associated with poor DSS and OS after RC in univariate analyses (Table 3). There was a significant association between sarcopenia status and T4 stage tumor (Table 2). Similar findings have previously been reported in patients with gastric cancer [13], with a higher rate of T3 and T4 among patients with a positive sarcopenia status at baseline. Body weight loss, a poor nutritional status and decreased Hb are known to be markers of cancer cachexia [25]. Elevation of inflammatory proteins, such as interleukin (IL)-6 and tumor necrosis factor alpha (TNF-α), may lead to various physical changes, including a decrease in muscle mass and adipose tissue in patients with sarcopenia.

Only a few reports have evaluated the clinical impact of a change in nutritional status and body composition after surgery and during oncological treatment. Previous studies have reported on changes in skeletal muscle mass during chemotherapy in patients with unresectable colorectal cancer [16] and advanced lung cancer [19], changes in skeletal muscle and adipose tissue in gastric cancer patients undergoing total gastrectomy [18] and changes in muscle, fat and bone mass during androgen blockade in prostate cancer patients [17, 26].

Notably, Hanson et al. provided evidence of the effectiveness of strength training in contributing to gains in muscle mass and strength, with these gains being associated with improved quality of life in patients with prostate cancer treated by androgen blockade [17]. Here, we hypothesized that decreases in the volume of the psoas major muscle and abdominal fat tissue, as well as nutritional status was associated with poor clinical outcomes, which could be improved with appropriate interventions. We identified a transient decrease in the volume of the psoas major muscle and abdominal fat tissue, as well as deterioration in the two nutritional indices, at 1 and 3 months after RC (Fig. 2). Interestingly, no postoperative decrease in the total area of abdominal skeletal muscles (sum of areas for the psoas major, paraspinal and abdominal wall muscles) was identified after RC (Fig. 2d). Therefore, targeted assessment of the psoas major muscle, postoperatively, could provide a proxy measure of physical deterioration after RC. Moreover, a postoperative loss in the volume of the psoas major muscle was identified as a strong prognostic factor of OS in our subanalysis among patients *with* and *without* sarcopenia (Fig. 3 and Figure S2). Our result was almost consistent with previous studies demonstrating the role of psoas muscle mass as a prognostic factor after RC [6, 7, 27].

Although a postoperative decrease in the PNI did not have prognostic value in the whole cohort, a decrease in PNI was identified as prognostic factor among patient *without* sarcopenia (Fig. 3b). The low baseline PNI among patients *with* sarcopenia could limit the postoperative change in reaching significant, providing a likely explanation for between-group differences with regards to the prognostic value of PNI. Moreover, there was a significant association of postoperative muscle loss and deterioration in nutrition status with a longer duration of hospitalization after RC (Fig. 3c-e). Therefore, efforts to prevent complications and shorten hospitalization period, as possible, are required to improve clinical outcomes after RC.

The limitations of our study need to be acknowledged in the interpretation of our results. These include data from a single-institution, the retrospective nature of the study, including the potential selection bias, and analysis with relatively small sample size and short follow-up duration. As well, definitions of muscle loss and deterioration in nutritional status, although used in previous studies, remain controversial. Optimal cutoffs need to be validated in an independent multi-institutional sample to establish a novel risk assessment tool specific to RC.

## Conclusions

To our knowledge this is the first study to have evaluated the clinical relevance of postoperative change in body composition, including skeletal muscle mass and nutritional status, as a predictor of clinical outcomes and survival after RC. Further research should identify appropriate rehabilitation protocols and nutritional interventions to maintain skeletal muscle mass and nutritional status, which could counteract deterioration in physical status and improve outcomes after RC.

## Additional files

Additional file 1: **Table S1.** Body composition index and nutrition index before radical cystectomy in 89 patients undergoing radical cystectomy. (DOCX 40 kb)

Additional file 2: **Figure S1.** Time course of change in the body composition and nutritional indices after radical cystectomy. For the body composition indices, the time course of change in absolute values is plotted. Data are expressed as means and standard deviations. Scores at each time point (1, 3, 6, 12, and 24 months after radical cystectomy) were compared to baseline (BL) scores using the Wilcoxon signed-rank test: *, *P* < 0.05; **, *P* < 0.01; and ***, *P* < 0.001. (TIFF 4267 kb)

Additional file 3: **Figure S2.** Comparison of changes after radical cystectomy between adjuvant chemotherapy-treated group and non-treated group.Time-course changes in cross-section area of the psoas major muscle at the level of L3 (a), abdominal skeletal muscle area at the level of L3 (b), the PNI (c) and, the CONUT score (d). Data of adjuvant chemotherapy-treated group (red) and non-treated group (blue) are plotted by means ± SD. Scores of two groups compared in each time point by the Mann-Whitney U-test. *ns*, not significant. (TIFF 7216 kb)

Additional file 4: **Figure S3.** Comparison of changes after radical cystectomy between neoadjuvant chemotherapy-treated group and non-treated group. Time-course changes in cross-section area of the psoas major muscle at the level of L3 (a), abdominal skeletal muscle area at the level of L3 (b), the PNI (c) and, the CONUT score (d). Data of neoadjuvant chemotherapy-treated group (red) and non-treated group (blue) are plotted by means ± SD. Scores of two groups compared in each time point by the Mann-Whitney U-test. *ns*, not significant. (TIFF 7424 kb)

Additional file 5: **Figure S4.** Kaplan-Meier curves for disease-specific survival and overall survival. Kaplan-Meier curves for disease-specific survival (DSS) and overall survival (OS) were compared for the postoperative change in the PNI (a), CONUT score (b), and psoas muscle mass (c). The cutoff values for postoperative change after RC was set based on median values as follows: −10%, −5 points and 2 points, respectively. The following separate analyzes were performed: all patients (left panels), patients *without* sarcopenia (middle panels), and patients with sarcopenia (right panels). Survival curves are compared using the log rank test. (TIFF 13821 kb)

## Abbreviations

BMI: Body mass index
CONUT: The Controlling Nutritional Status
CT: Computed tomography
DSS: Disease-specific survival
EWGSOP: The European Working Group on Sarcopenia in Older People
HR: Hazard ratio
IQR: Interquartile range
MIBC: Muscle invasive bladder cancer
OS: Overall survival
PNI: The prognostic nutritional index
RC: Radical cystectomy
SMI: Skeletal muscle index
T-chol: Total cholesterol
UCB: Urothelial carcinoma of the bladder
VSR: Visceral-to-subcutaneous adipose tissue volume ratio
