# Erratum to: Clinical impact of postoperative loss in psoas major muscle and nutrition index after radical cystectomy for patients with urothelial carcinoma of the bladder

**DOI:** 10.1186/s12885-017-3325-2

**Published:** 2017-05-22

**Authors:** Makito Miyake, Yosuke Morizawa, Shunta Hori, Nagaaki Marugami, Keiji Shimada, Daisuke Gotoh, Yoshihiro Tatsumi, Yasushi Nakai, Takeshi Inoue, Satoshi Anai, Kazumasa Torimoto, Katsuya Aoki, Nobumichi Tanaka, Kiyohide Fujimoto

**Affiliations:** 1 Department of Urology, Nara Medical University, 840 Shijo-cho, Kashihara-shi, Nara, 634-8522 Japan; 2 Department of Radiology, Nara Medical University, 840 Shijo-cho, Kashihara-shi, Nara, 634-8522 Japan; 3 Department of Pathology, Nara City Hospital, 1-50-1 Higashi kidera-cho, Nara-shi, Nara, 630-8305 Japan

## Erratum

After the publication of this work [1] it was noticed that in the PDF version of Table 3, the variables, ‘Sex’, ‘Clinical T’, ‘Clinical N’, ‘Variant histology’, ‘Neoadjuvant chemotherapy’, ‘Adjuvant chemotherapy’, and ‘Sarcopenia status’ were incorrectly centred. The original version of this article was updated with the correct table layout so these variables appear on the left.
