# Supplementary material for: Clinical impact of postoperative loss in psoas major muscle and nutrition index after radical cystectomy for patients with urothelial carcinoma of the bladder
Source: BMC Cancer. 2017 Mar 31;17:237. doi: 10.1186/s12885-017-3231-7 (PMC5374611; doi:10.1186/s12885-017-3231-7)
Supplement: Supplementary file 1 — Table S1. Body composition index and nutrition index before radical cystectomy in 89 patients undergoing radical cystectomy. (DOCX 40 kb) [file 12885_2017_3231_MOESM1_ESM.docx]

| **Additional Table S1. Body composition index and nutrition index before radical cystectomy in 89 patients undergoing radical cystectomy** | | | | |
| --- | --- | --- | --- | --- |
| **Variables** |  | **Median (IQR)** | **Mean ± SD** | **P value†** |
| **BMI (kg/m^2^)** |  | **22.7 (20.9 - 24.7)** | **23.1 ± 3.5** |  |
| **Male/Female** |  | **22.8 (20.9 - 24.7) / 22.2 (20.6 - 24.7)** | **23.1 ± 3.5 / 22.9 ± 3.5** | **0.87** |
| **Abdominal muscle index** |  |  |  |  |
| **SMI at L3 (cm^2^/m^2^)** |  | **51.4 (44.6 - 57.4)** | **51.0 ± 10.7** |  |
| **Male/Female** |  | **52.0 (47.4 - 57.8) / 45.0 (41.7 - 53.3)** | **52.1 ± 11.1 / 46.9 ± 7.6** | **0.015** |
| **Sarcopenia by Martin’s definition** |  |  |  |  |
| **No / Yes** |  | **67 / 22** | **-** |  |
| **%** |  | **75% / 25%** | **-** |  |
| **Psoas major muscle area at L3 (cm^2^/m^2^)** |  | **7.3 (5.6 - 8.3)** | **7.2 ± 1.9** |  |
| **Male/Female** |  | **7.4 (6.3 - 8.6) / 5.9 (4.9 - 7.2)** | **7.5 ± 1.9 / 6.0 ± 1.1** | **0.0008** |
| **Total psoas major muscle volume (cm^3^/m^2^)** |  | **105.9 (82.7 - 131.4)** | **110.1 ± 33.6** |  |
| **Male/Female** |  | **118.1 (100.1 - 134.7) / 83.2 (68.4 - 102.3)** | **117.2 ± 33.7 / 85.9 ± 19.8** | **0.003** |
| **Partial volume of the psoas major muscle (L3-L4) (cm^3^/m^2^)** |  | **45.9 (32.6 - 53.2)** | **45.0 ± 12.0** |  |
| **Male/Female** |  | **48.0 (38.0 - 55.3) / 31.9 (29.1 - 43.5)** | **47.7 ± 11.6 / 35.6 ± 8.3** | **0.001** |
| **Abdominal adipose tissue index** |  |  |  |  |
| **Visceral fat index (cm^3^/m^2^)** |  | **949 (602 - 1642)** | **1112 ± 704** |  |
| **Male/Female** |  | **952 (595 - 1722) / 946 (631 - 1465)** | **1131 ± 751 / 1054 ± 553** | **0.87** |
| **Subcutaneous fat index (cm^3^/m^2^)** |  | **867 (630 - 1195)** | **1031 ± 680** |  |
| **Male/Female** |  | **792 (594 - 1081) / 1617 (921 - 2099)** | **840 ± 441 / 1635 ± 937** | **0.003** |
| **Visceral to subcutaneous ratio (VSR)** |  | **1.1 (0.75 - 1.6)** | **1.2 ± 0.54** |  |
| **Male/Female** |  | **1.3 (0.91 - 1.7) / 0.58 (0.52 - 0.76)** | **1.3 ± 0.5 / 0.73 ± 0.41** | **<0.0001** |
| **PEF (L/min) in spirometry** |  |  |  |  |
|  |  | **455 (423 - 477)** | **435 ± 66** |  |
| **Male/Female** |  | **467 (446 - 481) / 319 (301 - 330)** | **466 ± 28 / 321 ± 29** | **<0.0001** |
| **Normal / Decline** |  | **40 / 49** | **-** |  |
| **%** |  | **46% / 54%** | **-** |  |
| **Nutrition index** |  |  |  |  |
| **PNI** |  | **47.0 (43.5 - 50.2)** | **46.6 ± 5.5** |  |
| **Male/Female** |  | **47.3 (43.9 - 50.6) / 47.0 (41.5 - 49.0)** | **46.9 ± 5.3 / 45.7 ± 6.3** | **0.38** |
| **CONUT score** |  | **2 (1 - 3)** | **2.2 ± 1.6** |  |
| **Male/Female** |  | **2 (1 - 3) / 3 (2 - 4)** | **2.0 ± 1.5 / 2.8 ± 1.8** | **0.16** |
| **IQR = interquartile range; SD = standard deviation; BMI = body mass index; SMI = skeletal mass index; PEF = peak expiratory flow; PNI = prognostic nutritional index; CONUT = controlling nutritional status; †, Comparison between male and female with Mann-Whitney *U* test; ‡ According to Tsukioka's definition for Japanese** | | | | |
