# Supplementary figures and images for: Clinical impact of postoperative loss in psoas major muscle and nutrition index after radical cystectomy for patients with urothelial carcinoma of the bladder
Source: BMC Cancer. 2017 Mar 31;17:237. doi: 10.1186/s12885-017-3231-7 (PMC5374611; doi:10.1186/s12885-017-3231-7)

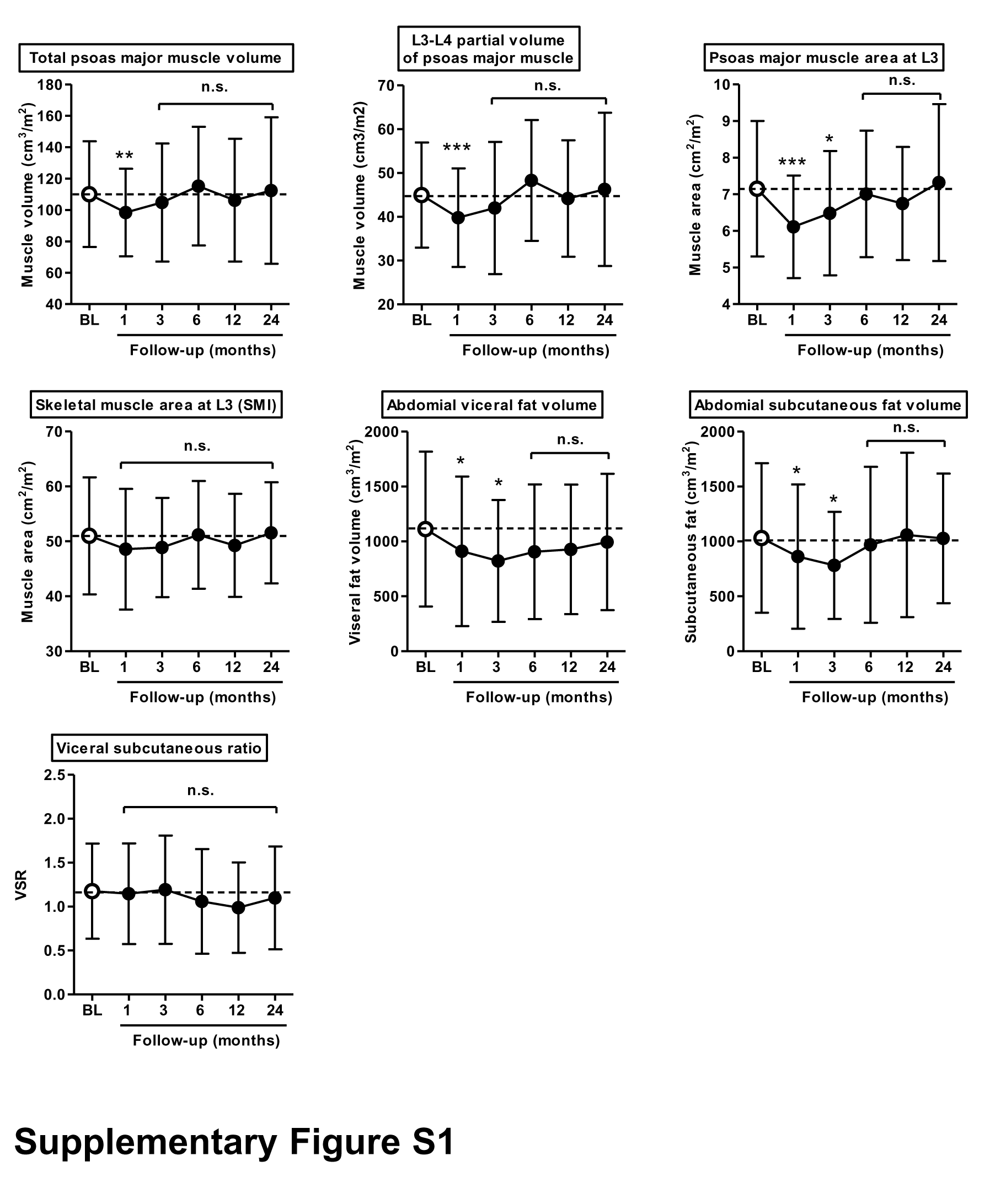

Supplement: Supplementary file 2 — Figure S1. Time course of change in the body composition and nutritional indices after radical cystectomy. For the body composition indices, the time course of change in absolute values is plotted. Data are expressed as means and standard deviations. Scores at each time point (1, 3, 6, 12, and 24 months after radical cystectomy) were compared to baseline (BL) scores using the Wilcoxon signed-rank test: *, P < 0.05; **, P < 0.01; and ***, P < 0.001. (TIFF 4267 kb) [file 12885_2017_3231_MOESM2_ESM.tif]

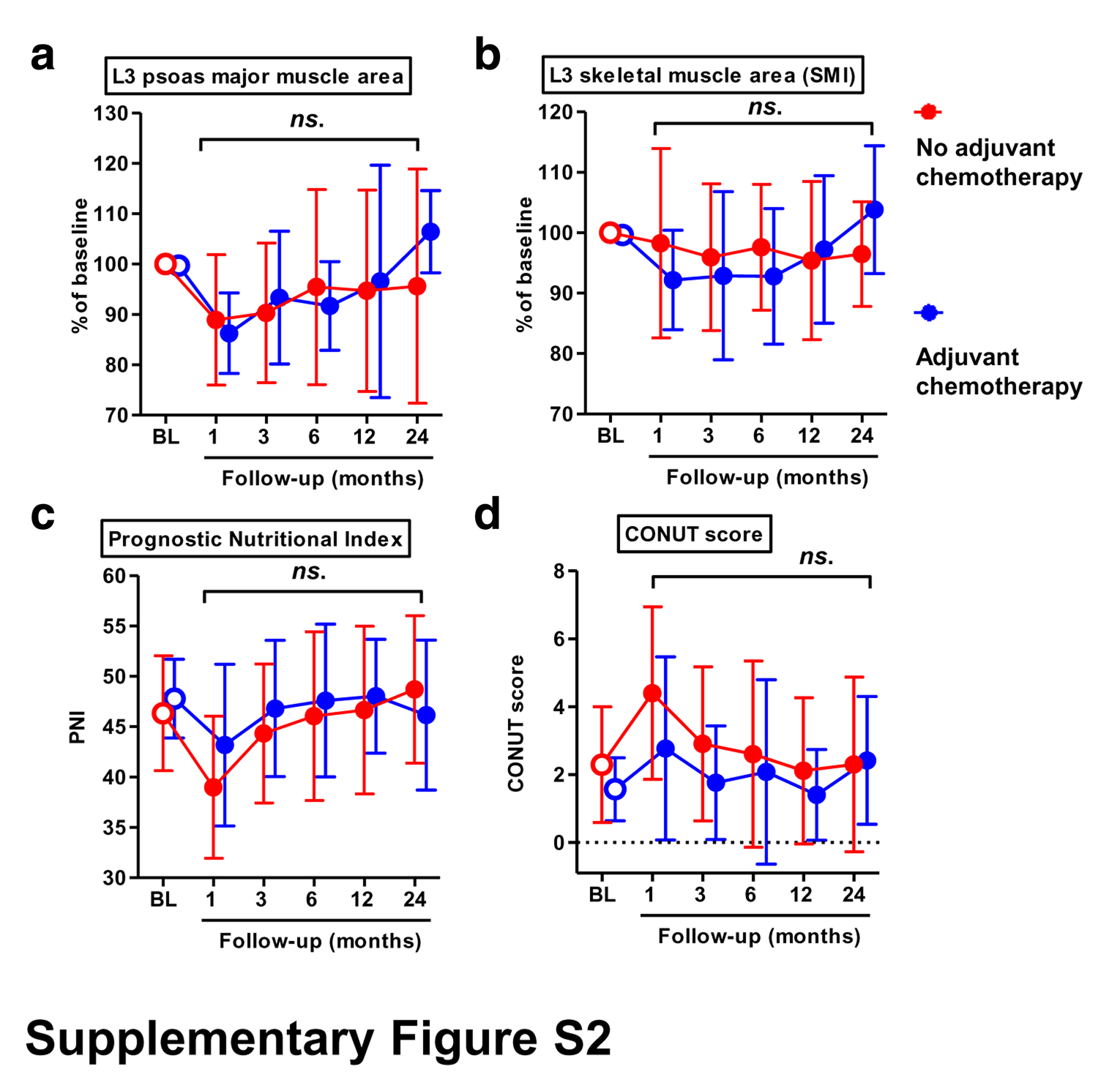

Supplement: Supplementary file 3 — Figure S2. Comparison of changes after radical cystectomy between adjuvant chemotherapy-treated group and non-treated group.Time-course changes in cross-section area of the psoas major muscle at the level of L3 (a), abdominal skeletal muscle area at the level of L3 (b), the PNI (c) and, the CONUT score (d). Data of adjuvant chemotherapy-treated group (red) and non-treated group (blue) are plotted by means ± SD. Scores of two groups compared in each time point by the Mann-Whitney U-test. ns, not significant. (TIFF 7216 kb) [file 12885_2017_3231_MOESM3_ESM.tif]

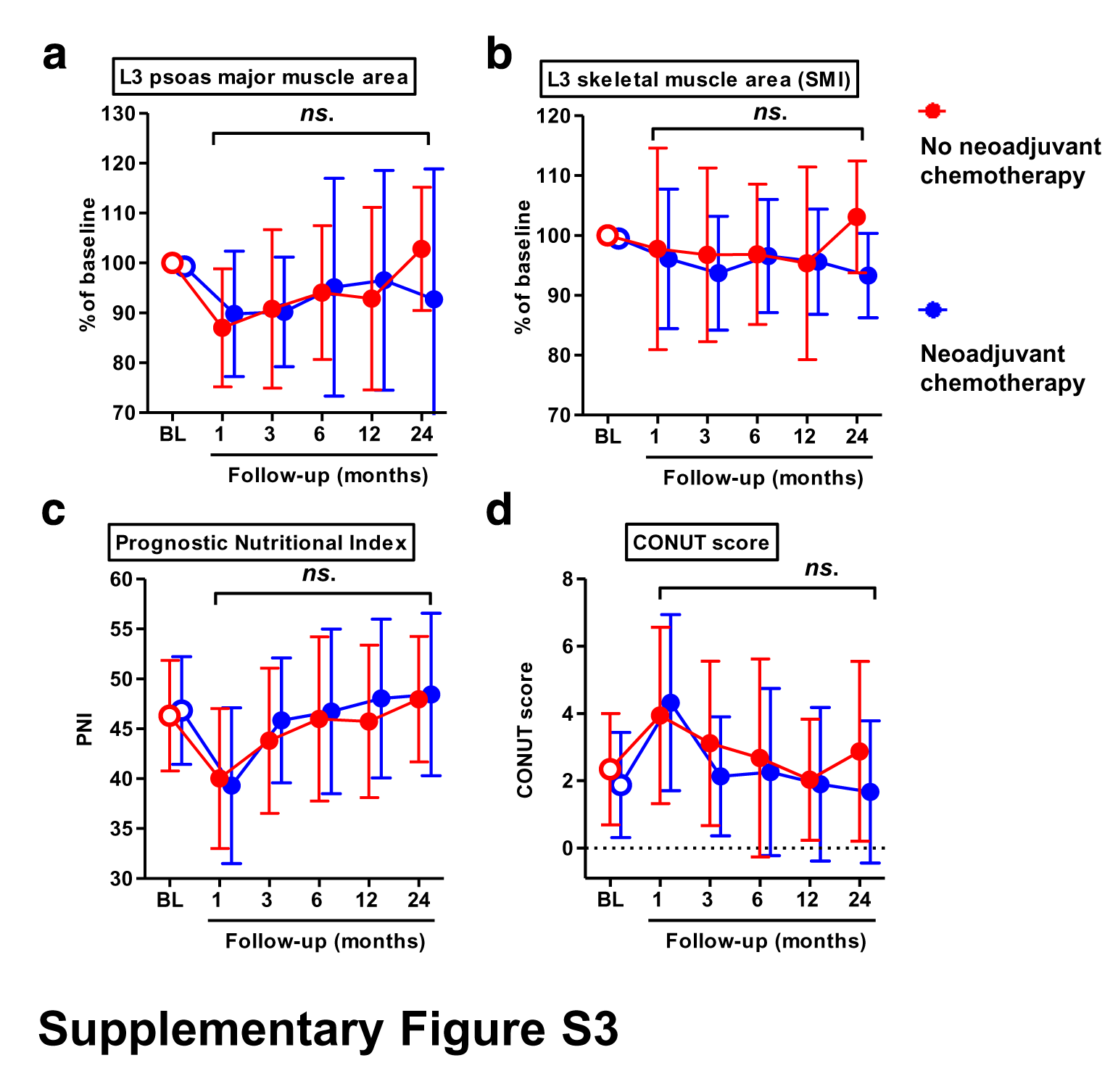

Supplement: Supplementary file 4 — Figure S3. Comparison of changes after radical cystectomy between neoadjuvant chemotherapy-treated group and non-treated group. Time-course changes in cross-section area of the psoas major muscle at the level of L3 (a), abdominal skeletal muscle area at the level of L3 (b), the PNI (c) and, the CONUT score (d). Data of neoadjuvant chemotherapy-treated group (red) and non-treated group (blue) are plotted by means ± SD. Scores of two groups compared in each time point by the Mann-Whitney U-test. ns, not significant. (TIFF 7424 kb) [file 12885_2017_3231_MOESM4_ESM.tif]

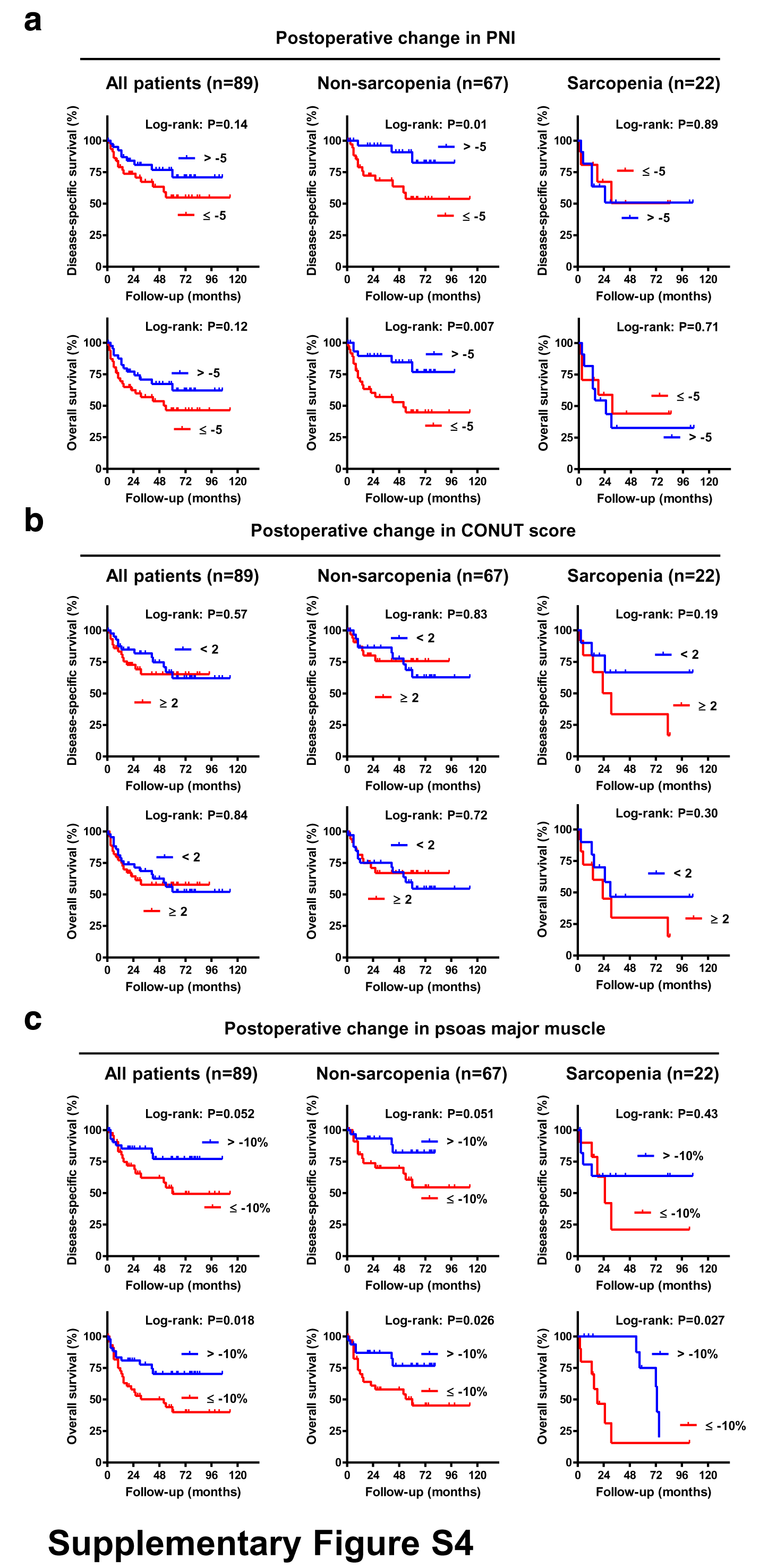

Supplement: Supplementary file 5 — Figure S4. Kaplan-Meier curves for disease-specific survival and overall survival. Kaplan-Meier curves for disease-specific survival (DSS) and overall survival (OS) were compared for the postoperative change in the PNI (a), CONUT score (b), and psoas muscle mass (c). The cutoff values for postoperative change after RC was set based on median values as follows: −10%, −5 points and 2 points, respectively. The following separate analyzes were performed: all patients (left panels), patients without sarcopenia (middle panels), and patients with sarcopenia (right panels). Survival curves are compared using the log rank test. (TIFF 13821 kb) [file 12885_2017_3231_MOESM5_ESM.tif]
